# Supplementary material for: The interactome of intact mitochondria by cross-linking mass spectrometry provides evidence for coexisting respiratory supercomplexes
Source: Mol Cell Proteomics. 2017 Dec 8;17(2):216–32. doi: 10.1074/mcp.RA117.000470 (PMC5795388; doi:10.1074/mcp.RA117.000470)
Supplement: Supplemental Data [file supp_RA117.000470_133922_0_supp_23978_fzffwf.zip › spectra_annotation/mito_DR_spectra_annotation/336-2-12-1-2-1.pdf]

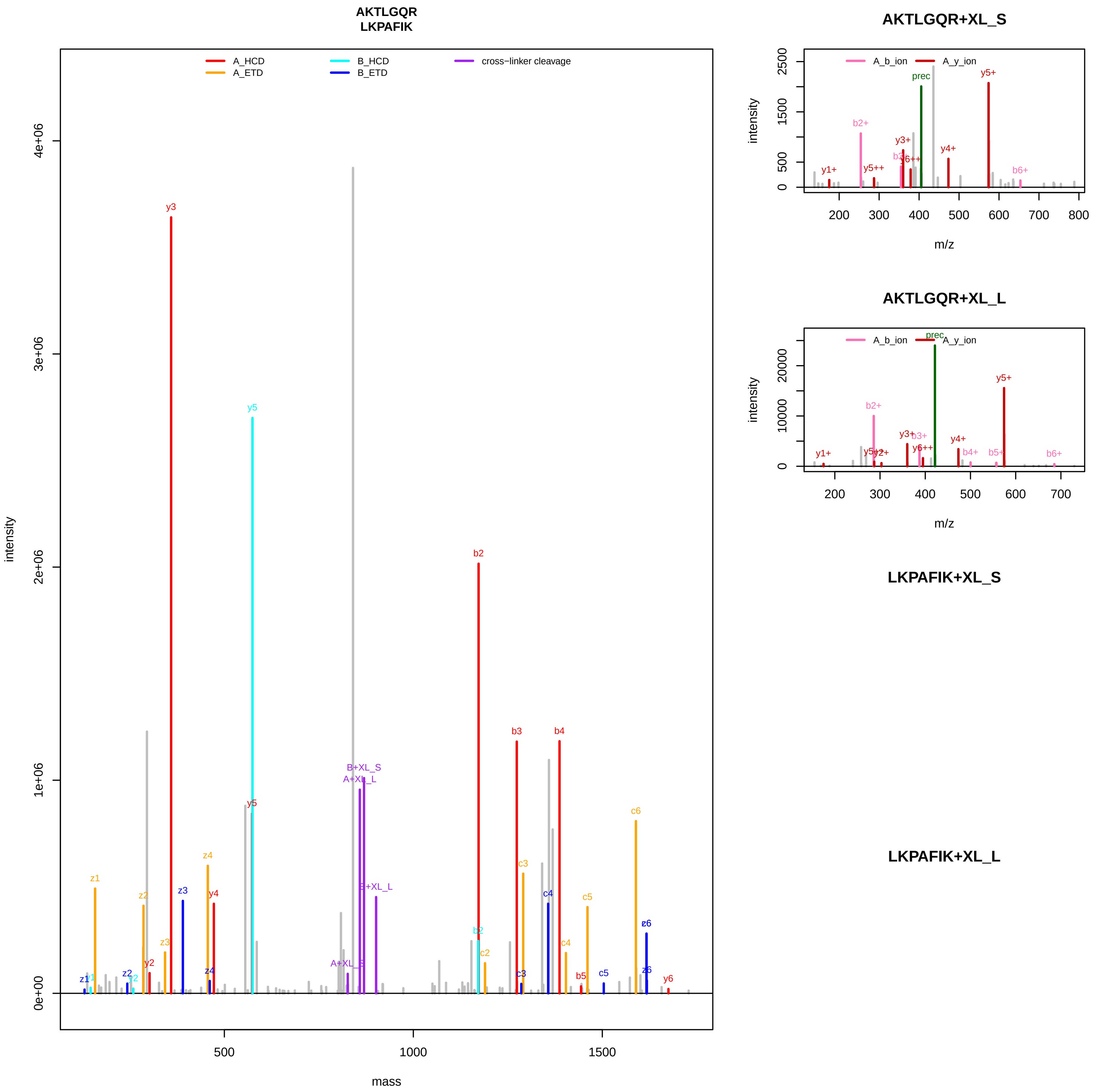
AKTLGQR
LKPAFIK
A_HCD
A_ETD
B_HCD
B_ETD
cross−linker cleavage
intensity
mass
0e+00
1e+06
2e+06
3e+06
4e+06
500
1000
1500
y3
y5
b2
b3
b4
c6
B+XL_S
A+XL_L
B+XL_L
A+XL_S
z1
z2
z3
z4
y2
y4
c2
c3
c4
c5
b5
y6
z6
AKTLGQR+XL_S
A_b_ion
A_y_ion
prec
y1+
y5++
b2+
y3+
y6++
y4+
y5+
b6+
m/z
AKTLGQR+XL_L
b3+
b4+
b5+
LKPAFIK+XL_S
LKPAFIK+XL_L
